# “RYR1 and the cerebellum”: scientific commentary on “Defective Cerebellar Ryanodine Receptor Type 1 and Endoplasmic Reticulum Calcium ‘Leak’ in Tremor Pathophysiology”

**DOI:** 10.1007/s00401-024-02687-0

**Published:** 2024-02-07

**Authors:** Heinz Jungbluth, Dennis T. Famili, Rick C. Helmich, Stefano Previtali, Nicol C. Voermans

**Affiliations:** 1 Department of Paediatric Neurology, Neuromuscular Service, Evelina London Children’s Hospital, Guy’s and St. Thomas’ Hospital NHS Foundation Trust, Children’s Neurosciences Centre, F02 – Becket House, Lambeth Palace Road, London, SE1 7EU UK; 2 Randall Centre for Cell and Molecular Biophysics, Muscle Signalling Section, Faculty of Life Sciences and Medicine (FoLSM), King’s College London, London, UK; 3 Department of Neurology, Radboud University Medical Centre, Nijmegen, The Netherlands; 4 Neuromuscular Repair Unit, Division of Neuroscience, IRCCS Ospedale San Raffaele, Milan, Italy

We read with interest the article entitled “Defective ryanodine receptor type 1 and endoplasmic reticulum calcium ‘leak’ in tremor pathophysiology” recently published in Acta Neuropathologica (2023 August;146(2): 301–318) [1]. In this work, Martuscello and colleagues implicate abnormal modification and functioning of the cerebellar Purkinje cell-expressed type 1 ryanodine receptor (RyR1) in essential tremor (ET), a very common neurological syndrome that is strongly linked to abnormal oscillatory activity in the cerebellum [2]. Moreover, the authors were also able to replicate the tremor phenotype in a mouse model mimicking post-translational RyR1 modifications seen in ET patients, and, more importantly, to ameliorate the murine phenotype through the administration of pharmacological compounds specifically aimed at stabilizing ‘leaky’ RyR1 channels.

We believe that for several reasons these findings are highly relevant beyond their immediate importance for the understanding of ET pathophysiology:

1. The *RYR1* gene encodes the principal skeletal muscle sarcoplasmic reticulum (SR) calcium release channel (RyR1) with a crucial role in excitation–contraction coupling. Despite its ubiquitous expression and its relevance for calcium signalling in non-skeletal muscle tissues including the brain, currently recognized *RYR1*-associated disorders are largely limited to primary neuromuscular conditions with a very wide clinical spectrum, ranging from severe early-onset congenital myopathies [3] to induced episodic phenotypes such as malignant hyperthermia (MH) and (exertional) rhabdomyolysis (ERM) [4, 5] in otherwise healthy individuals. Implication of RyR1 in a brain disorder like ET widens this clinical spectrum beyond the neuromuscular domain, in line with reports suggesting a role of RyR1 in an increasing number of non-skeletal muscle presentations such as mild bleeding disorders [6] and, more recently, pancreatitis [7].
2. The findings by Martuscello et al. confirm a specific vulnerability of cerebellar Purkinje cells to RyR1 dysfunction, as already suggested by our earlier observation of fatal cerebellar swelling in a case with *RYR1*-related MH and marked Purkinje cells abnormalities on post mortem examination [8]. Moreover, heat-induced CNS injury appears to have a predilection for the cerebellum [9], an observation which so far has remained poorly understood, but which may well at least partly be explained by the mechanisms described in the present paper. Additional information regarding the specific *RYR1* genotypes of the ET patients included in this study would have been of interest in this context, considering that the unequivocal RyR1 alterations observed in the reported ET cohort remain unaccounted for but may well have been due to primary alterations of the *RYR1* gene in these patients.
3. In support of the hypothesis above, we recently identified another family with a *RYR1*-related myopathy and additional cerebellar involvement. These two siblings, a brother and a sister currently in their 60 s, developed moderate muscle weakness pronounced proximally with some additional peroneal involvement from late childhood. In addition, they developed a cerebellar syndrome characterized by ataxia, horizontal nystagmus and saccadic eye movements from adolescence. CK levels were moderately increased at 500 IU/l, and EMG was suggestive of proximal myogenic and distal neurogenic features. Brain MRI performed in both siblings showed cerebellar atrophy (Fig. 1). In addition to other non-specific features, muscle biopsy in the brother demonstrated increased fibre size variability, increased central nuclei and central cores on oxidative stains compatible with a *RYR1*-related myopathy. Whole exome sequencing (WES) revealed compound heterozygosity for *RYR1* variants p.(Arg224Alafs*6) and p.(Arg3772Gln), the latter a likely pathogenic MH mutation (www.emhg.org). No other genetic cause for the cerebellar syndrome was identified on WES.

**Fig. 1:**
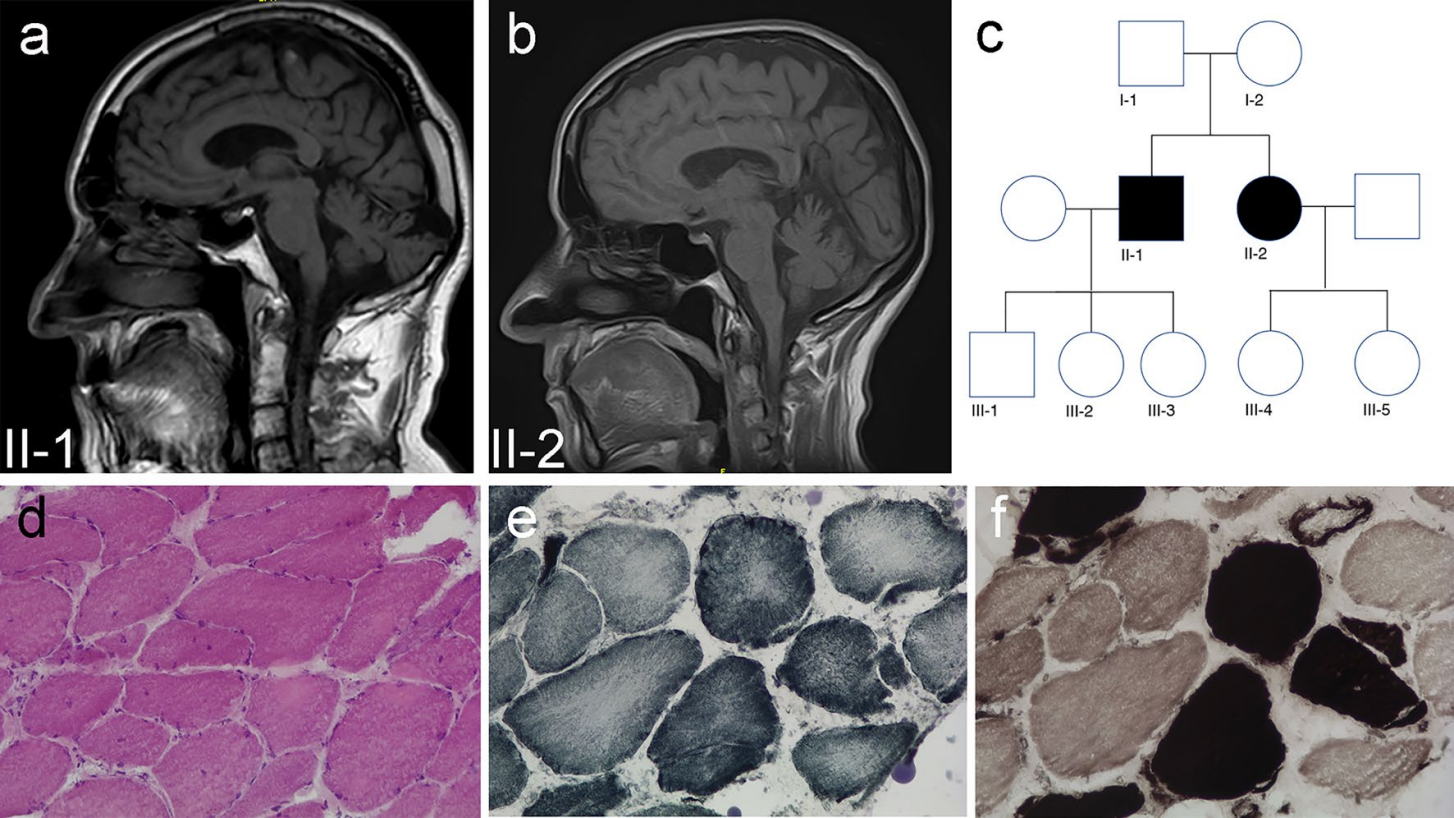
Cerebellar involvement in patients with a *RYR1*-related disorder. Brain MRI, sagittal T1-weighted images (**a**, **b**), demonstrating cerebellar atrophy in a brother (II-1) and sister (II-2) from a non-consanguineous family (**c**) with a cerebellar syndrome on the background of a moderate, predominantly proximal myopathy due to compound heterozygosity for *RYR1* variants p.(Arg224Alafs*6) and p.(Arg3772Gln). Muscle biopsy (**d**–**f**) showed increased variability in fibre size with an increased number of centralized nuclei and centrally pronounced unevenness of oxidative stains in different fibre types (**d** H&E; **e** NADH-TR; **f** ATPase 4.6)

Taken together, these observations suggest a role of altered RyR1 receptor function not only in skeletal muscle disorders but also probably in a much wider range of neurological and other conditions. Most of the non-skeletal muscle-associated phenotypes reported to date were associated with (putative) *RYR1* gain-of-function variants, but the full genotypical spectrum of such presentations has yet to be elucidated. Pharmacological compounds aimed at correcting abnormal RyR1 function may be of benefit not only in *RYR1*-related neuromuscular disorders but potentially also a wider range of non-skeletal muscle disorders.

## Data Availability

Original data included in this letter are available on reasonable request.
